# Toward understanding microbiota homeostasis in the plant kingdom

**DOI:** 10.1371/journal.ppat.1009472

**Published:** 2021-04-22

**Authors:** Bradley C. Paasch, Sheng Yang He

**Affiliations:** 1 Department of Energy Plant Research Laboratory, Michigan State University, East Lansing, Michigan, United States of America; 2 Department of Biochemistry and Molecular Biology, Michigan State University, East Lansing, Michigan, United States of America; 3 Department of Biology, Duke University, Durham, North Carolina, United States of America; 4 Howard Hughes Medical Institute, Durham, North Carolina, United States of America; THE SAINSBURY LABORATORY, UNITED KINGDOM

## Abstract

A diverse community of microorganisms inhabits various parts of a plant. Recent findings indicate that perturbations to the normal microbiota can be associated with positive and negative effects on plant health. In this review, we discuss these findings in the context of understanding how microbiota homeostasis is regulated in plants for promoting health and/or for preventing dysbiosis.

## Defining the “core” microbiota in plants

In the past decade, many studies have surveyed microbiota composition in plants. Despite diverse microbes found in plants, overall bacterial composition seems to be conserved at the phylum level. In flowering plants that have been surveyed, the aboveground tissues (phyllosphere) are associated with bacterial assemblages dominated by Proteobacteria, Actinobacteria, Bacteroidetes, and Firmicutes. These bacterial phyla are also enriched in belowground tissues (rhizosphere) compared to bulk soil [1,2]. Nonvascular plants such as liverwort and moss are also dominated by these phyla [3–5], suggesting possible conservation of core microbiota across plant lineages. It should be pointed out that, so far, most microbiota surveys have been focused on bacterial components of plant-associated microbial communities. As fungi [6], viruses [7], and protozoa [8] are also common residents on or inside the plant, more efforts are needed in the future to systematically define fungal, viral, and protozoa microbiota members across plant taxa. Additionally, biogeography plays an important role in determining the reservoir of microbes from which a plant selects members of its microbiota [9]. Because different microbial taxa may provide functionally redundant traits to a plant host [10], detection of dissimilar taxa in different plants, especially at lower taxonomical levels, may not necessarily indicate functionally distinct microbiotas, an important topic that requires rigorous future investigations.

Regardless of whether there is a functionally conserved core microbiome in plants, increasing evidence suggests that microbiota homeostasis may be intimately linked to host processes and, in turn, plant health. Microbiota homeostasis in eukaryotic organisms is likely dynamic in both space and time. Here, we use eubiosis to describe the state of microbiota homeostasis that is necessary for maintaining host health under optimal, nonstressful conditions. The eubiotic state for an individual plant is not static, but instead dynamic over a plant’s lifetime. For example, microbiota of healthy plants can vary temporally based on the time of year [11] or developmental stage [12]. Stress or other perturbations may induce changes to the microbiota which could disrupt eubiosis. Disruption to microbiota eubiosis can be associated with negative impacts on host health and is often called dysbiosis in this context [13]. Deviation from eubiosis, however, is not always detrimental and may help plants cope with various forms of stress. Below, we focus our discussion on the interplay between microbiota homeostasis and plant health and resilience.

## Microbiota homeostasis shifts toward dysbiosis

In humans, dysbiosis is associated with ailments such as inflammatory bowel disease, diabetes, allergies, and other health issues [14] and is often accompanied by a lower diversity microbial community with altered metabolic function [15]. However, broad use of the term “dysbiosis” in mammalian literature has come under scrutiny, particularly for its inconsistent definition and ambiguity as often no distinction can be made between it being a cause or effect of a specific disease [16]. A recent study showed an example of dysbiosis as the causal agent for tissue damages in plants. Several *Arabidopsis* immune-compromised mutants were found to harbor an increased amount and altered composition of phyllosphere microbiota and display leaf-tissue damage under high humidity [17]. The Shannon diversity index and the relative abundance of Firmicutes were markedly reduced, whereas Proteobacteria were enriched inside the leaves of these mutant plants, bearing cross-kingdom resemblance to some aspects of the dysbiosis that occurs in human inflammatory bowel disease. Importantly, bacterial community transplantation experiments showed that the application of the dysbiotic leaf bacterial community to otherwise healthy plants resulted in tissue damages, demonstrating that, in this case, dysbiosis is causative to negative impact on host health [17].

Tissue damage-associated microbiota shifts have also been observed during insect and pathogen attacks, which often compromise host immune responses. For example, herbivory of bittercress plants by the leaf-mining fly causes a significant shift in phyllosphere microbiota, resulting in an increased abundance of bacteria on damaged leaves. Growth of *Pseudomonas* spp. (belonging to Proteobacteria) was found to largely account for the increased abundance of microbiota [18]. Another study found that fungal pathogen *Zymoseptoria tritici* suppresses immune responses in susceptible wheat cultivars, resulting in an increase in bacteria members of the leaf microbiome near fungal infection sites [19]. Together, these examples imply that immune suppression during pathogen infections is associated with shifting the composition of microbiota, similar to what is observed in immune-compromised plant mutants [17]. This may be a broadly applicable principle. Indeed, microbiota changes have been described across many plant species upon biotic challenge, including citrus greening in citrus [20], parasitic nematode *Meloidogyne graminicola* infection in rice [21], Yellow Canopy Syndrome in sugarcane [21], and protist *Plasmodiophora brassicae* in Chinese cabbage [22]. However, in these cases, it is not yet known whether the observed changes in microbiota contribute causally to (or a consequence of) tissue damages in disease.

## Microbiota homeostasis shifts benefiting plant stress resilience

Stress-induced deviation from eubiosis is not always associated with reduced plant performance. Microbiota changes (referred to hereafter as meliorbiosis; from the Latin root melior*-* meaning “to make better, improve”) that enable positive effects on plant performance under stressful conditions have been described. In the case of biotic stress, the fungal pathogen *Fusarium graminearum* was shown to induce shifts to rhizosphere microbiota of barley plants, including apparent recruitment of bacterial taxa that are enriched with antifungal traits [23]. In *Arabidopsis*, infection of leaves with oomycete *Hyaloperonospora arabidopsidis*, a causative agent of downy mildew, resulted in enrichment of specific rhizosphere bacteria that were able to induce systemic resistance against downy mildew [24]. Insect herbivory can also induce changes to the plant microbiota. For example, aphid [25] and whitefly [25,26] feeding of aboveground pepper plant tissues results in restructuring of rhizosphere microbiota and enhanced resilience to belowground bacterial pathogens.

Abiotic stress can also induce microbiota shifts. Drought stress, for instance, induces a large restructuring of belowground communities across diverse plant hosts [27–29]. This shift is generally associated with enrichment of Actinobacteria in the root endosphere relative to the rhizosphere or bulk soil [28]. The enrichment of specific strains under drought conditions, but not water replete conditions, is correlated with increased plant root biomass [29] which could contribute to improved drought resilience [30].

As is in the case of dysbiosis, the cause-and-effect relationship during meliorbiosis is still not so clear in many cases. While changes in microbiota composition are sometimes associated with positive effects on plant fitness, causality still needs to be demonstrated in most instances.

## Plant factors regulating microbiota homeostasis

**Fig 1:**
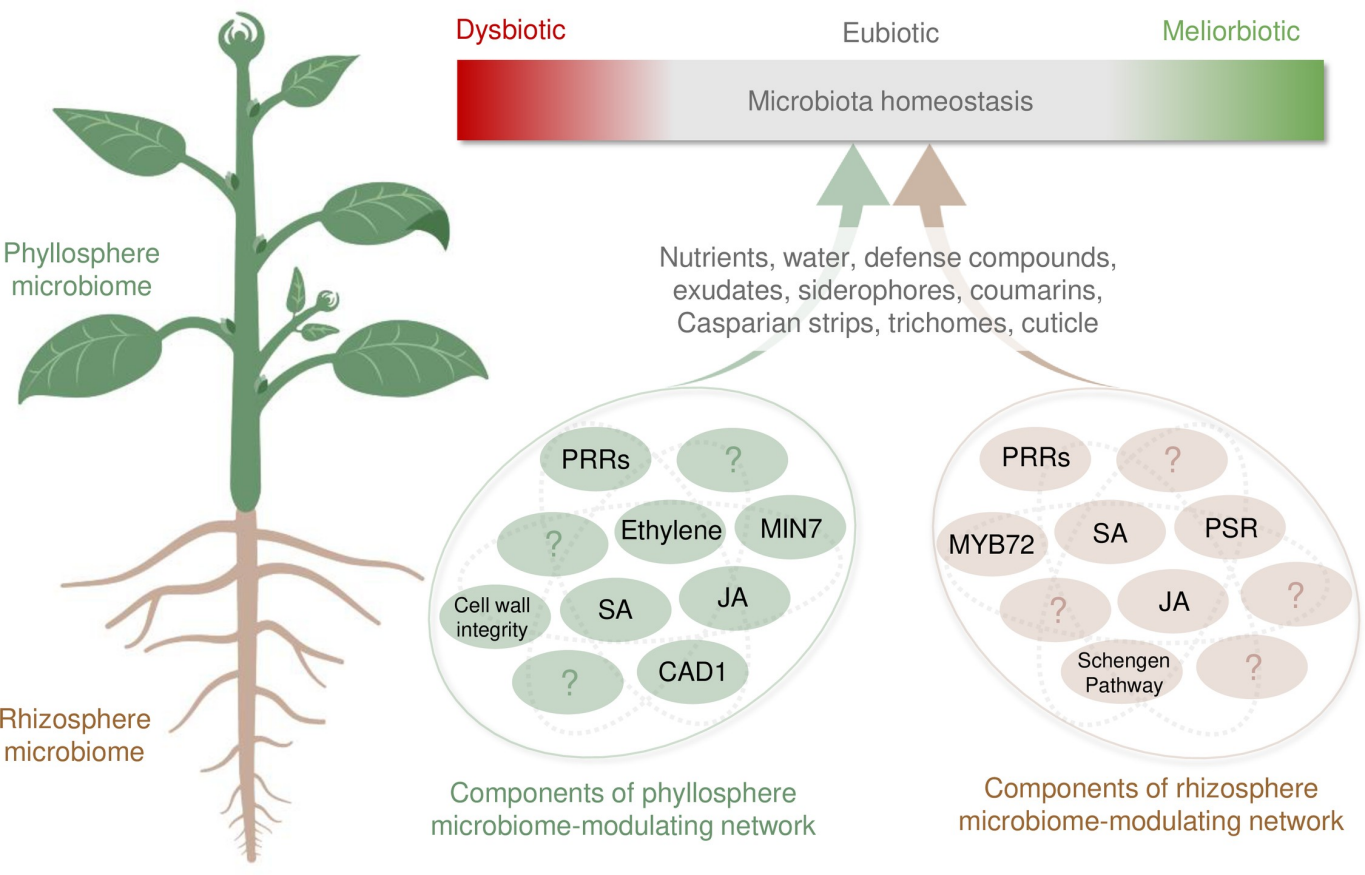
Host control of microbiota homeostasis in plants. Microbiota eubiosis represents a normal range of microbiota abundance and composition in healthy plants grown under optimal conditions. If eubiosis is disrupted, either by host mutations, abiotic stress, or infections, or a combination thereof, homeostasis can shift toward a dysbiotic state associated with negative impacts on plant health or toward a meliorbiotic state associated with positive impacts on plant health. Examples of host factors that contribute to microbiota homeostasis in the phyllosphere (green) and rhizosphere (brown) are depicted in circles below. Not all known factors are depicted. CAD1, constitutively activated cell death 1; JA, jasmonic acid; MIN7, HOPM1-interactor 7; MYB72, MYB domain protein 72; PRRs, pattern recognition receptors; PSR, phosphate starvation response; SA, salicylic acid. Created with Biorender.

If proper microbiota homeostasis is critical for plant health, one would expect that plants would have evolved mechanisms to prevent health-damaging dysbiosis and allow health-promoting meliorbiosis under stressful conditions. Indeed, recent studies have begun to identify host factors that are involved in mediating microbiome homeostasis in plants (Fig 1). While it is well established that plant defense hormones salicylic acid (SA) and jasmonic acid (JA) play an important role in limiting the growth of virulent pathogens in plants, it is becoming increasingly evident that they also play a critical role in mediating the homeostasis of commensal microbiota members. *Arabidopsis* mutants with constitutively elevated levels of SA-mediated immune response harbor reduced bacterial diversity in the endophytic leaf microbiota, while mutants deficient in JA-mediated immune response harbor an increased bacterial diversity in epiphytic leaf microbiota [31]. Activation of JA pathways by application of methyl-JA also alters the composition of the rhizosphere microbiota [32], further implicating the role of JA pathways in the regulation of microbiome homeostasis in plants. A study involving multiple hormone mutants identified defense hormone SA is required to establish a normal rhizosphere microbiota and that SA-mediated modulation of the rhizosphere microbiota is likely to occur at the family level, instead of impacting only a select few largely abundant strains [33]. In addition to SA and JA, *Arabidopsis ein2* mutants defective in ethylene signaling harbor distinct phyllosphere microbial communities compared to wild-type plants [34]. However, it remains to be determined whether the observed microbiota alterations in these defense hormone mutants causally impact plant fitness, either positively or negatively, an area of great interest for future research.

In addition to defense hormones, recent studies have begun to show a critical role of pattern-triggered immunity (PTI) in regulating modulating microbiota homeostasis in plants [17,35,36]. PTI is a major form of plant immunity initiated by plant recognition of common microbial patterns by pattern-recognition receptors (PRRs) [37]. An *Arabidopsis* quadruple mutant lacking 3 PRRs/coreceptors (recognizing bacterial flagellin, elongation factor Tu, and peptidoglycan, respectively) and a vesicle traffic regulator, the MIN7 protein, displayed a dysbiotic shift in the quantity and composition of the endophytic leaf microbiota [38]. Similar alterations in endophytic leaf microbiota were found in an *Arabidopsis* mutant that carries a S205F mutation in a membrane attack complex/perforin domain protein, CAD1 [17]. The involvement of plant immunity in regulating some aspects of microbiota homeostasis illustrates a conceptual parallel to mammalian-microbiome interactions, as PRR gene mutations (e.g., NOD2) have been shown to be linked to dysbiosis in humans [39] and members of the MACPF protein family, such as human C9 and perforin in particular, have been shown to be involved in innate and adaptive immunity in mammals [40].

In addition to defense-related plant processes, physical barriers and leaf-surface structures such as the plant cell wall and trichomes may play a role in influencing microbiota homeostasis based on a genome-wide association study examining 196 accessions of *Arabidopsis* grown in the field and their associated bacterial and fungal communities [41]. Furthermore, *Arabidopsis* mutants with altered cuticle formation possess altered epiphytic phyllosphere bacterial communities [34,42]. A recent study further confirmed the role of physical structures in contributing to microbiota homeostasis [43]. Here, *Arabidopsis* mutants defective in genes controlling the function of endodermal root diffusion barriers, including those in the Schengen pathway required for Casparian strip formation and those involved with suberin deposition, possessed rhizosphere bacterial communities with altered composition [43].

Finally, pathways involved in plant nutrient response also play a role in microbiota homeostasis. For instance, under iron- and phosphate-limiting conditions, plants can induce the secretion of coumarins which, in addition to aiding in plant nutrient uptake, possess selective antimicrobial activity and can shape the root microbiota [44]. *Arabidopsis*, for instance, secretes iron-mobilizing coumarin scopoletin under iron-limiting conditions in response to beneficial bacteria in a manner dependent on the MYB72 transcription factor and a β-glucosidase, BGLU42. Scopoletin was found to have high antimicrobial activity against fungal pathogens *Fusarium oxysporum* and *Verticillium dahlia*, whereas many beneficial rhizobacteria are tolerant [45]. Additionally, several *Arabidopsis* mutants defective in components regulating phosphate starvation response (PSR) and inorganic phosphate availability in plant tissues were found to harbor endophytic root microbial communities that are significantly different compared to wild-type plants [46].

## Future outlook

In this review, we have highlighted several examples of environmental and host influences on microbiota homeostasis in plants, a topic that is likely to become intensively studied in the coming decade. However, the current understanding is rather limited, and the cause–effect relationship has not been resolved in most cases. Further advances will likely hinge on progress in two critical areas. First, application of multiple methodologies will be needed to go beyond taxonomic knowledge of microbiota to gain insight into functional implications of microbiota shifts as traits rather than taxa may be selected during microbiome shifts. The application of methodologies such as metagenomics, metatranscriptomics, or metabolomics could inform on the functional implication of a particular microbiome. Further, incorporation of quantitative taxonomic analyses, either by direct quantification, DNA spike-in, or normalization to host DNA (i.e., chloroplasts and mitochondria as described in [18]), for instance, could help identify ecologically relevant fine-scale differences between microbial communities that may not be apparent in current studies.

Second, it is now time to more clearly define the taxonomical and functional features of eubiosis, dysbiosis, and meliorbiosis in plants and, perhaps more importantly, to resolve the cause–effect relationship between observed microbiota shifts and plant fitness. While methodologies, such as shotgun metagenomics, may enable researchers to generate hypotheses related to microbiome function, we anticipate increased use of rationally designed synthetic microbial communities [47] and innovative gnotobiotic plant growth systems [48] in future research in this area. Indeed, this is already happening [17,49–53], and we can expect significant progress in the next few years to reach a better understanding of how microbiota homeostasis is controlled in plants. Broad understanding of microbiota homeostasis should provide a critical knowledge base for the development of innovative plant- and/or microbiota-based solutions to globally improve pre- and post-harvest plant health and productivity.

## References

[1] Müller DB, Vogel C, Bai Y, Vorholt JA. The Plant Microbiota: Systems-Level Insights and Perspectives. Annu Rev Genet. 2016;50:211–34. 10.1146/annurev-genet-120215-034952 27648643

[2] Bulgarelli D, Schlaeppi K, Spaepen S, van EVL, Structure S-LP. Functions of the Bacterial Microbiota of Plants. Annu Rev Plant Biol. 2013:807–38. 10.1146/annurev-arplant-050312-120106 23373698

[3] Alcaraz LD, Peimbert M, Barajas HR, Dorantes-Acosta AE, Bowman JL, Arteaga-Vázquez MA. Marchantia liverworts as a proxy to plants’ basal microbiomes. Sci Rep. 2018;8:12712. 10.1038/s41598-018-31168-0 30140076 PMC6107579

[4] Bragina A, Oberauner-Wappis L, Zachow C, Halwachs B, Thallinger GG, Müller H, et al. The Sphagnum microbiome supports bog ecosystem functioning under extreme conditions. Mol Ecol. 2014;23:4498–510. 10.1111/mec.12885 25113243

[5] Ma J, Tang JY, Wang S, Chen ZL, Li XD, Li YH. Illumina sequencing of bacterial 16S rDNA and 16S rRNA reveals seasonal and species-specific variation in bacterial communities in four moss species. Appl Microbiol Biotechnol. 2017;101:6739–53. 10.1007/s00253-017-8391-5 28664321

[6] Bergelson J, Mittelstrass J, Horton MW. Characterizing both bacteria and fungi improves understanding of the Arabidopsis root microbiome. Sci Rep. 2019;9:24. 10.1038/s41598-018-37208-z 30631088 PMC6328596

[7] Roossinck MJ. A new look at plant viruses and their potential beneficial roles in crops. Mol Plant Pathol. 2015:331–3. 10.1111/mpp.12241 25904282 PMC6638535

[8] Gao Z, Karlsson I, Geisen S, Kowalchuk G, Jousset A. Protists: Puppet Masters of the Rhizosphere Microbiome. Trends Plant Sci. 2019;24:165–76. 10.1016/j.tplants.2018.10.011 30446306

[9] Fitzpatrick CR, Salas-González I, Conway JM, Finkel OM, Gilbert S, Russ D, et al. The Plant Microbiome: From Ecology to Reductionism and Beyond. Annu Rev Microbiol. 2020;74:81–100. 10.1146/annurev-micro-022620-014327 32530732

[10] Louca S, Polz MF, Mazel F, Albright MBN, Huber JA, O’Connor MI, et al. Function and functional redundancy in microbial systems. Nat Ecol Evol. 2018;2:936–43. 10.1038/s41559-018-0519-1 29662222

[11] Grady KL, Sorensen JW, Stopnisek N, Guittar J, Shade A. Assembly and seasonality of core phyllosphere microbiota on perennial biofuel crops. Nat Commun. 2019;10:4135. 10.1038/s41467-019-11974-4 31515535 PMC6742659

[12] Chaparro JM, Badri DV, Vivanco JM. Rhizosphere microbiome assemblage is affected by plant development. ISME J. 2014;8:790–803. 10.1038/ismej.2013.196 24196324 PMC3960538

[13] Petersen C, Round JL. Defining dysbiosis and its influence on host immunity and disease. Cell Microbiol. 2014;16:1024–33. 10.1111/cmi.12308 24798552 PMC4143175

[14] Blum HE. The human microbiome. Adv Med Sci. 2017;62:414–20. 10.1016/j.advms.2017.04.005 28711782

[15] DeGruttola AK, Low D, Mizoguchi A, Mizoguchi E. Current Understanding of Dysbiosis in Disease in Human and Animal Models. Inflamm Bowel Dis. 2016;22:1137–50. 10.1097/MIB.0000000000000750 27070911 PMC4838534

[16] Hooks KB, O’Malley MA. Dysbiosis and Its Discontents. MBio. 2017;8. 10.1128/mBio.01492-17 29018121 PMC5635691

[17] Chen T, Nomura K, Wang X, Sohrabi R, Xu J, Yao L, et al. A plant genetic network for preventing dysbiosis in the phyllosphere. Nature. 2020;580:653–7. 10.1038/s41586-020-2185-0 32350464 PMC7197412

[18] Humphrey PT, Whiteman NK. Insect herbivory reshapes a native leaf microbiome. Nat Ecol Evol. 2020;4:221–9. 10.1038/s41559-019-1085-x 31988447 PMC7332206

[19] Seybold H, Demetrowitsch TJ, Hassani MA, Szymczak S, Reim E, Haueisen J, et al. A fungal pathogen induces systemic susceptibility and systemic shifts in wheat metabolome and microbiome composition. Nat Commun. 2020;11:1910. 10.1038/s41467-020-15633-x 32313046 PMC7171108

[20] Trivedi P, He Z, Van JD, Albrigo G, Zhou J, Wang N. Huanglongbing alters the structure and functional diversity of microbial communities associated with citrus rhizosphere. ISME J. 2012;6:363–83. 10.1038/ismej.2011.100 21796220 PMC3260493

[21] Hamonts K, Trivedi P, Garg A, Janitz C, Grinyer J, Holford P, et al. Field study reveals core plant microbiota and relative importance of their drivers. Environ Microbiol. 2018;20:124–40. 10.1111/1462-2920.14031 29266641

[22] Lebreton L, Guillerm-Erckelboudt A-Y, Gazengel K, Linglin J, Ourry M, Glory P, et al. Temporal dynamics of bacterial and fungal communities during the infection of Brassica rapa roots by the protist Plasmodiophora brassicae. PLoS ONE. 2019;14:e0204195. 10.1371/journal.pone.0204195 30802246 PMC6388920

[23] Dudenhöffer J, Scheu S, Jousset A. Systemic enrichment of antifungal traits in the rhizosphere microbiome after pathogen attack. J Ecol. 2016:1566–75. 10.1111/1365-2745.12626

[24] Berendsen RL, Vismans G, Yu K, Song Y, de R, Burgman WP, et al. Disease-induced assemblage of a plant-beneficial bacterial consortium. ISME J. 2018;12:1496–507. 10.1038/s41396-018-0093-1 29520025 PMC5956071

[25] Lee B, Lee S, Ryu C-M. Foliar aphid feeding recruits rhizosphere bacteria and primes plant immunity against pathogenic and non-pathogenic bacteria in pepper. Ann Bot. 2012;110:281–90. 10.1093/aob/mcs055 22437662 PMC3394643

[26] Yang JW, Yi H-S, Kim H, Lee B, Lee S, Ghim S-Y, et al. Whitefly infestation of pepper plants elicits defence responses against bacterial pathogens in leaves and roots and changes the below-ground microflora. J Ecol. 2011:46–56. 10.1111/j.1365-2745.2010.01756.x

[27] Santos-Medellín C, Edwards J, Liechty Z, Nguyen B, Sundaresan V. Drought Stress Results in a Compartment-Specific Restructuring of the Rice Root-Associated Microbiomes. MBio. 2017. 10.1128/mbio.00764-17 28720730 PMC5516253

[28] Fitzpatrick CR, Copeland J, Wang PW, Guttman DS, Kotanen PM, Johnson MTJ. Assembly and ecological function of the root microbiome across angiosperm plant species. Proc Natl Acad Sci U S A. 2018;115:E1157–65. 10.1073/pnas.1717617115 29358405 PMC5819437

[29] Xu L, Naylor D, Dong Z, Simmons T, Pierroz G, Hixson KK, et al. Drought delays development of the sorghum root microbiome and enriches for monoderm bacteria. Proc Natl Acad Sci U S A. 2018;115:E4284–93. 10.1073/pnas.1717308115 29666229 PMC5939072

[30] Fang Y, Xiong L. General mechanisms of drought response and their application in drought resistance improvement in plants. Cell Mol Life Sci. 2015;72:673–89. 10.1007/s00018-014-1767-0 25336153 PMC11113132

[31] Kniskern JM, Traw MB, Bergelson J. Salicylic acid and jasmonic acid signaling defense pathways reduce natural bacterial diversity on Arabidopsis thaliana. Mol Plant-Microbe Interact. 2007;20:1512–22. 10.1094/MPMI-20-12-1512 17990959

[32] Carvalhais LC, Dennis PG, Badri DV, Tyson GW, Vivanco JM, Schenk PM. Activation of the jasmonic acid plant defence pathway alters the composition of rhizosphere bacterial communities. PLoS ONE. 2013;8:e56457. 10.1371/journal.pone.0056457 23424661 PMC3570460

[33] Lebeis SL, Paredes SH, Lundberg DS, Breakfield N, Gehring J, McDonald M, et al. Salicylic acid modulates colonization of the root microbiome by specific bacterial taxa. Science. 2015;349:860–4. 10.1126/science.aaa8764 26184915

[34] Bodenhausen N, Bortfeld-Miller M, Ackermann M, Vorholt JA. A synthetic community approach reveals plant genotypes affecting the phyllosphere microbiota. PLoS Genet. 2014;10:e1004283. 10.1371/journal.pgen.1004283 24743269 PMC3990490

[35] Song Y, Wilson AJ, Zhang X-C, Thoms D, Sohrabi R, Song S, et al. Loss of a plant receptor kinase recruits beneficial rhizosphere-associated Pseudomonas. BioRxiv [Preprint]. 2020; [posted 2020 Nov 02; cited 2020 Dec 27]. 10.1101/2020.11.02.364109

[36] Ma K-W, Niu Y, Jia Y, Ordon J, Copeland C, Emonet A, et al. Coordination of microbe-host homeostasis via a crosstalk with plant innate immunity. Research Square [Preprint]. 2020; [posted 2020 Sep 10; cited 2020 Dec 27]. 10.21203/rs.3.rs-69445/v1

[37] Zipfel C. Pattern-recognition receptors in plant innate immunity. Curr Opin Immunol. 2008:10–6. 10.1016/j.coi.2007.11.003 18206360

[38] Xin X-F, Nomura K, Aung K, Velásquez AC, Yao J, Boutrot F, et al. Bacteria establish an aqueous living space in plants crucial for virulence. Nature. 2016;539:524–9. 10.1038/nature20166 27882964 PMC5135018

[39] Lauro ML, Burch JM, Grimes CL. The effect of NOD2 on the microbiota in Crohn’s disease. Curr Opin Biotechnol. 2016;40:97–102. 10.1016/j.copbio.2016.02.028 27035071 PMC5820027

[40] Liu X, Lieberman J. Knocking ‘em Dead: Pore-Forming Proteins in Immune Defense. Annu Rev Immunol. 2020:455–85. 10.1146/annurev-immunol-111319-023800 32004099 PMC7260445

[41] Horton MW, Bodenhausen N, Beilsmith K, Meng D, Muegge BD, Subramanian S, et al. Genome-wide association study of Arabidopsis thaliana leaf microbial community. Nat Commun. 2014;5:5320. 10.1038/ncomms6320 25382143 PMC4232226

[42] Reisberg EE, Hildebrandt U, Riederer M, Hentschel U. Distinct phyllosphere bacterial communities on Arabidopsis wax mutant leaves. PLoS ONE. 2013;8:e78613. 10.1371/journal.pone.0078613 24223831 PMC3818481

[43] Salas-González I, Reyt G, Flis P, Custódio V, Gopaulchan D, Bakhoum N, et al. Coordination between microbiota and root endodermis supports plant mineral nutrient homeostasis. Science. 2020. 10.1126/science.abd0695 33214288

[44] Voges MJEEE, Bai Y, Schulze-Lefert P, Sattely ES. Plant-derived coumarins shape the composition of an synthetic root microbiome. Proc Natl Acad Sci U S A. 2019;116:12558–65. 10.1073/pnas.1820691116 31152139 PMC6589675

[45] Stringlis IA, Yu K, Feussner K, de R, Van S, Van MC, et al. MYB72-dependent coumarin exudation shapes root microbiome assembly to promote plant health. Proc Natl Acad Sci U S A. 2018;115:E5213–22. 10.1073/pnas.1722335115 29686086 PMC5984513

[46] Castrillo G, Teixeira PJPL, Paredes SH, Law TF, de L, Feltcher ME, et al. Root microbiota drive direct integration of phosphate stress and immunity. Nature. 2017;543:513–8. 10.1038/nature21417 28297714 PMC5364063

[47] Vorholt JA, Vogel C, Carlström CI, Müller DB. Establishing Causality: Opportunities of Synthetic Communities for Plant Microbiome Research. Cell Host Microbe. 2017;22:142–55. 10.1016/j.chom.2017.07.004 28799900

[48] Kremer JM, Sohrabi R, Paasch BC, Rhodes D, Thireault C, Schulze-Lefert P, et al. Peat-based gnotobiotic plant growth systems for Arabidopsis microbiome research. Nat Protoc. 2021. Forthcoming. 10.1038/s41596-021-00504-6 PMC10354810 33911260

[49] Durán P, Thiergart T, Garrido-Oter R, Agler M, Kemen E, Schulze-Lefert P, et al. Microbial Interkingdom Interactions in Roots Promote Arabidopsis Survival. Cell. 2018;175:973, e14–83. 10.1016/j.cell.2018.10.020 30388454 PMC6218654

[50] Finkel OM, Salas-González I, Castrillo G, Spaepen S, Law TF, Teixeira PJPL, et al. The effects of soil phosphorus content on plant microbiota are driven by the plant phosphate starvation response. PLoS Biol. 2019;17:e3000534. 10.1371/journal.pbio.3000534 31721759 PMC6876890

[51] Carlström CI, Field CM, Bortfeld-Miller M, Müller B, Sunagawa S, Vorholt JA. Synthetic microbiota reveal priority effects and keystone strains in the Arabidopsis phyllosphere. Nat Ecol Evol. 2019;3:1445–54. 10.1038/s41559-019-0994-z 31558832 PMC6774761

[52] Herrera Paredes S, Gao T, Law TF, Finkel OM, Mucyn T, Teixeira PJPL, et al. Design of synthetic bacterial communities for predictable plant phenotypes. PLoS Biol. 2018;16:e2003962. 10.1371/journal.pbio.2003962 29462153 PMC5819758

[53] Finkel OM, Salas-González I, Castrillo G, Conway JM, Law TF, Pereira PJ, et al. A single bacterial genus maintains root growth in a complex microbiome. Nature. 2020:103–8. 10.1038/s41586-020-2778-7 32999461 PMC10329457

